# Effectiveness of Teaching Emotion Regulation Strategies in Improving Cognitive-Emotional Regulation Among Female Students in Addiction-Stricken Areas of Kermanshah City 

**Published:** 2018-12

**Authors:** Afsaneh Shahbazirad, Mona Azizi

**Affiliations:** Substance Abuse Prevention Research Center, Kermanshah University of Medical Sciences, Kermanshah, Iran

**Keywords:** Emotion Regulation Strategies, Cognitive-Emotional Regulation, Adolescent, Addiction

## Abstract

**Objective:** To evaluate emotion regulation as one of the important aspects of preventing and treatment of substance abuse.

**Materials and methods:** This study had a quasi-experiment research method and a pretest-posttest design with the control group. The statistical population of this study included all the female students who lived in the addiction-stricken areas of Kermanshah city. 60 female students (mean: 16.78 and standard deviation: 0.69) who were eligible to participate in the study were selected by convenient sampling and were placed randomly in two test and control groups. The tools that were used in this study included Granefski et al. (2007) cognitive-emotional regulation scale and demographic information sheet. Eight weekly sessions of emotion regulation strategies based on Gross method (2002) were held for the experimental group.

**Results:** Multivariate covariance analysis results indicated that teaching emotion regulation strategies has been effective in improving adapting strategies and reducing maladaptive strategies of emotion regulation (p < 0.05).

**Conclusion:** Therefore considering the effectiveness of the mentioned intervention, this strategy can be applied beside other methods in order to improve adaptive emotion regulation and reducing maladaptive strategies among female teenagers who live in addiction-stricken areas.

## Introduction

Using drugs is one of the biggest issues in many countries, but drug addiction as a special situation is a relatively new phenomenon that has been recognized since late 18^th^ and early 19^th^ century (1). Several psychological, social, familial and biological factors play roles in drug dependency and addiction (2). Difficulty in regulating emotions as a psychological factor is a common problem among drug-addicted people that leads to failure in managing their state of affection and emotion (3). People who are unable to control their emotional skills are more likely to use addictive drugs in order to face their negative emotions (4). Cognitive regulation of emotions is considered a process through which people moderate their emotions in response to conscious and unconscious environmental demands and is a particular kind of self-adjustment. As a whole, emotion regulation is one the main factors of wellbeing and successful activism and plays an important role in coping with stressful events of life (5).

When an individual is under pressure of his peers to use drugs, effective management of emotions reduces the risk for drug abuse (6). Various approaches have been introduced regarding teaching emotion regulation among which Grossmodel is famous and includes 5stages (beginning, situation, attention, assessment and response). Every stage includes a series of adaptive strategies and a series of maladaptive strategies and individuals who have emotional problems are more likely to use maladaptive strategies such as rumination, avoidance etc. Thus, by intervention in emotional problems, we should modify or eliminate maladaptive strategies and teach adaptive ones (7). The form of emotion regulation strategies is so that based on it, the positive emotions increase and the negative emotions are reduced or eliminated. Moreover, emotions too will be moderated in this process; in a way that with a monitoring system, excessive increase or decrease in emotions will be controlled. This system of emotion regulation is developed and improved since childhood and will eventually help the individual improve his ability to control emotional information processing and have a more successful individual and social life. Therefore, emotion regulation is a complex and multi-dimensional phenomenon that occurs by coordinating many biological and behavioral processes (8, 9, 10).

Few studies have been conducted regarding the effectiveness of emotion regulation strategies in improving cognitive regulation of emotion among which we can refer to Borjali et al. (11), Sadri Damirchi et al. (12), Hasani and Shahmoradifar (13) and Thghizadeh et al. (14) that suggested emotion regulation strategies teaching has an effect on the reduction of negative emotions. 

Addiction and drug abuse is a social issue in which the ability of the society for organizing and retaining the existing order diminishes and the regular function of social life is interrupted (15). As drug abuse and addiction to various kinds of drugs and their consequences are considered big social problems around the world (16), the importance of having preventing strategies is becoming evident more than ever. Studies show that proper treatment can considerably reduce the risk of dangerous behaviors such as addiction, suicide and teenager escape before and after the incident. Nevertheless, only a small percent of these people undergo treatment and because of the risky situation of these people, few studies have been conducted in order to find proper and organized treatments with controlled framework for the reduction of suicide, escape and substance abuse risks among this population (17). Because of their curious personality and their going through adolescence and the mental crisis of this stage, teenagers are considered one of the most vulnerable groups of the society against crimes and consequences related to drugs and addiction. Pardini et al. suggested that improper coping methods play a moderating role in the relation between negative emotions (e.g. anger and sadness) and drugs abuse. Thus, angry teenagers use drugs as a way of coping with their problems (18). Considering that early experiences of using drugs or having addicted parents can make people vulnerable to addiction and that various researches have confirmed this (19, 20), as a result prevention programs must be aimed towards at-risk groups and work towards the improvement and teaching of proper strategies and educational interventions to prevent this group from addiction. Moreover, since the adolescent group, particularly the female ones are at high risk and considering the strategic position of Kermanshah City which is located in the borders of the country and the point that the beginning of substance abuse goes back to school years, also regarding that different studies have stressed the emotional and affective defects among addicts which is considered an important factor in the tendency towards drugs, and also because of the research gap about teaching emotion regulation strategies to high-risk groups, it is necessary to improve cognitive regulation among female adolescents by teaching emotion regulation strategies so they learn how to deal with their own problems and issues and be less likely to use maladaptive and ineffective methods such as drugs abuse. Therefore, this study tries to examine whether teaching emotion regulation strategies can be effective in improving cognitive-emotional regulation among the female teenagers of addiction-stricken areas of Kermanshah City.

## Materials and methods

This work was a quasi-experimental pretest-posttest study with the control group. The statistical population of this study included all the female students of addiction-stricken areas of Kermanshah who were studying in the second half of the year 2017. In order to selected people, at the beginning, one area was selected randomly from among the high-risk areas of the city and then from this area, two all-girl high schools were picked out at random, from which one was randomly selected as experimental group and another was selected as control group. Following that, 60 students who tended to participate did not have a record of psychosis and mental illness were selected and placed into the experimental and control group. Finally, educational intervention of emotion regulation strategies based on Gross model (8 ninety-minute sessions) was administered for the experimental group and both of the experimental and control groups were evaluated and measured by the above-mentioned scale. The following ethical aspects were considered in this study: The participants were free to choose to be part of this study and had given their written consent. The participants were assured that their individual private information would be kept confidential and would be reported collectively. Thedata were categorized and summarized using descriptive statistics indices. The inferential statistics indices including multi-variate covariance analysis were also used to analyze the data. The software considered for analyzing the data was SPSS-19. The following tools were used for information gathering:

***Cognitive-emotional regulation scale:*** this scale has been developed by Granefski et al. It has 36 questions which are answered based on Likert rating in a range of never (1) to always (5).This tool is comprised of 9 sub-scales every one of which measures a particular cognitive strategy for emotion regulation using 4 questions. These strategies include self-blame, other-blame, acceptance, refocus on planning, positive refocus, rumination, positive reappraisal, putting into perspective and catastrophizing (21). Psychometric indices of this scale have been predicted as 0.78 and 0.83 for any of those subscales using Cronbach alpha (22). The short version of this scale which has 18 questions has been used in this study.

***Demographic information sheet*** *:* it included age and major of study. 

***Therapy Protocols for Emotion Regulation Strategies***

*First session:* group members make each other's acquaintance, describing the rationale and intervention stages and the framework and the instructions for participating in the group.

*Second sessions:* recognizing emotions and stimulating situations through teaching the difference of function between various emotions and the short-term and long-term effects of emotions.

*Third session:* assessing the extent of vulnerability and the emotional skills of the members

*Forth session:* making change in the emotion-stimulating situation and teaching inter-personal skills (conversation, assertion and conflict solving).

*Fifth session:* redirecting attention and stopping rumination and worrying

*Sixth session:* changing the cognitive assessment and teaching reappraisal strategies

*Seventh session:* changing the behavioral and physiological consequences of emotion

*Eighth session:* reappraisal and removing practical barriers.

## Results

Our sample included 60 female adolescents with an average age of 16.73 ± 0.63in the experimental group and 16.83 ± 0.74 in the control group. The results of descriptive indices of the adaptive strategies were (pre-test: 17.22, post-test 21.33) in the experimental group and (pre-test: 17.04, post-test: 16.98) in the control group. Moreover, the results obtained for descriptive indices of the maladaptive strategies were (pre-test 15.84, post-test: 10.47) in the experimental group and (pre-test: 15.49, post-test: 15.30) in the control group (Table 1).

In order to do the covariance analysis, at first the scores normality assumptions and the homogeneity of variances were examined and for that purpose, Kolmogorov-Smirnov Test and Levene’s Test were applied. Kolmogorov-Smirnov Test results in the pre-test and post-test of the adaptive strategies were obtained as (o.39, 0.59) and the results of this test in the pre-test and post-test of the maladaptive strategy were calculated as (0.80, 0.82).

**Table1:** Results of multivariate Covariance analysis

Variables	Test	Value	F	Error df	Hypothesis df	Significant level	Eta effect
**Group**	Pillai’s Trace	0.711	67.50	55	2	0.001	0.711
	Wilks’ Lambda	0.289	67.50	55	2	0.001	0.711
	Hotelling’s Trace	2.45	67.50	55	2	0.001	0.711
	Roy’s Largest Root	2.45	67.50	55	2	0.001	0.711

**Table 2:** Results of univariate Covariance analysis

Dependant variable	Sum of Square	df	Mean square	F	Significant level	Eta effect	Observed power
**Adaptive strategy**	271.27	1	271.27	46.38	0.001	0.45	1
**Maladaptive strategy**	365.50	1	365.50	103.17	0.001	0.45	1

Considering the statistical insignificance (0.1, 0.6, 0.13, 0.8) in Levene’s Test, it can be concluded that the homogeneity of the variances was present about the variables. The results of Box Test for examining equality of covariance matrix of the dependent variables between the test and the control group indicated that the covariance matrices of the dependent variables are equal in both groups (p > 0.05, F= 13.59, F = 3.73). Chi-Square Bartlet test for the examination of the sphericity or the significance of the relationship between the variables indicated that there is a significant relationship between these variables (X^2 ^= 17.15, p < 0.05). After examining the assumptions for the multivariate covariance analysis , the results of the test showed that there is a meaningful difference between the two group of the variables (p < 0.001, F= 67.50), in order to determine in which variables the test and the control group differ, the results of the univariate variance analysis has been reported in table 2.

According to table2, the statistics of F for the adaptive strategies variables (46.38) is significant at the 0.001 level and for maladaptive strategies (103.17) it is significant at the level of 0.001.These findings suggest that there is a significant difference between the groups with regards to those variables. The results for examining the means in the following table show that the mean for adaptive strategies in the experimental group (21.29) is higher than the mean for the control group (17.02), and the mean for maladaptive strategies in the experimental group (10.41) is lower than the mean of the control group (15.36). The value of the effect of Eta shows that 45 percent of adaptive strategy variance and 64 percent of maladaptive strategy variance have been explained by emotion regulation strategies.

## Discussion

This study has been conducted in order to investigate the effectiveness of teaching emotion regulation strategies on improving cognitive-emotional regulation among female adolescents in addiction-stricken areas of Kermanshah. The results indicated that emotion regulation strategies have been effective in improving the adaptive strategies of cognitive emotional regulation. The results of this study are consistent with the studies by Borjali et al. (11), Sadri Damirchi et al. (12), Hasani and Shahmordaifar (13) and Thghizadeh et al. (14). The strategies of cognitive-emotional regulation are the ways through which the individual copes with the stressful situations (23, 24). Cognitive-emotional regulation is in a way like the cognitive management of the emotionally arousing information (25, 26). Therefore it can be suggested that emotion regulation teaching accompanied by teaching emotional self-control and self-management can teach people to strengthen themselves both emotionally and mentally, so that they have a higher ability to predict the expectations of others, show more resistance against the unexpected pressures by others and consequently resist more against drug abuse and have less tendency for drugs. Teaching the mentioned intervention would also lead to more awareness of others feeling, affective balance and more focus on programming in situations which in a way enables people to reappraise situations and conditions, and also have a better cognitive focus and positive reappraisal in solving their problems and issues.

The results also showed that emotion regulation strategies have been effective in reducing the maladaptive strategies of cognitive-emotional regulation. The results of this study have been consistent with the studies conducted by Borjali et al. (11) and Hasani and Shahmoradifar (13). In emotion regulation teaching, technics such as identifying maladaptive beliefs, rumination and worrying will be taught to the individuals which makes them evaluate their emotional responses and as a result they would choose adapting beliefs and strategies and become more flexible in response to their emotions and thus be less likely to use drugs in the face of stressful situations. Individuals who recognize their own negative feelings, regulate their emotional experience in a more effective way and thus will be more successful in coping and adapting to negative experiences. 

This study has been merely conducted about female adolescents; as a result one should be more cautious in generalizing the results. A lack of follow-up stage for further assessment of the efficiency of emotion regulation teaching, a lack of full control on the unwanted variables such as personal variables and mental state (memory, intelligence, tendency and …) of the participants are some other limitations of this study.

## Conclusion

As emotion regulation is one of the important aspects in preventing drug abuse and considering the effectiveness of the mentioned intervention, it is recommended that education authorities and school counselors take advantage of this technic by teaching emotion regulation strategy and take major steps in improving self-regulation among students and adolescents through the teaching of this strategy so that they develop more control and self-mastery methods in the face of stressful events.
